# Biocontrol potential of native isolates of *Beauveria bassiana* against cotton leafworm *Spodoptera litura* (Fabricius)

**DOI:** 10.1038/s41598-023-35415-x

**Published:** 2023-05-23

**Authors:** Shah Mohammad Naimul Islam, Md. Zahid Hasan Chowdhury, Mahjabin Ferdaous Mim, Milia Bente Momtaz, Tofazzal Islam

**Affiliations:** 1 Institute of Biotechnology and Genetic Engineering, Bangabandhu Sheikh Mujibur Rahman Agricultural University, Gazipur, Bangladesh; 2 Cotton Research Training and Seed Multiplication Farm, Gazipur, Bangladesh

**Keywords:** Biotechnology, Microbiology

## Abstract

The entomopathogenic fungus (EPF), *Beauveria bassiana*, is reported as the most potent biological control agent against a wide range of insect families. This study aimed to isolate and characterize the native *B. bassiana* from various soil habitats in Bangladesh and to evaluate the bio-efficacy of these isolates against an important vegetable insect pest, *Spodoptera litura*. Seven isolates from Bangladeshi soils were characterized as *B. bassiana* using genomic analysis. Among the isolates, TGS2.3 showed the highest mortality rate (82%) against the 2nd instar larvae of *S. litura* at 7 days after treatment (DAT). This isolate was further bioassayed against different stages of *S. litura* and found that TGS2.3 induced 81, 57, 94, 84, 75, 65, and 57% overall mortality at egg, neonatal 1st, 2nd, 3rd, 4th, and 5th instar larvae, respectively, over 7 DAT. Interestingly, treatment with *B. bassiana* isolate TGS2.3 resulted in pupal and adult deformities as well as decreased adult emergence of *S. litura*. Taken together, our results suggest that a native isolate of *B. bassiana* TGS2.3 is a potential biocontrol agent against the destructive insect pest *S. litura*. However, further studies are needed to evaluate the bio-efficacy of this promising native isolate *in planta* and field conditions.

## Introduction

The reduction of crop losses due to insects is becoming a bigger challenge for the world's food production. Due to concerns about their impact on human health, the environment, and the food chain, many of the older, less expensive chemical insecticides are no longer being registered^1^. New technologies like expensive, more selective chemicals and genetic modification are being used, but this increased selection pressure accelerates the evolution of resistance in insect pests. Global agriculture urgently needs more environmentally friendly pest management techniques.

The tobacco caterpillar, *Spodoptera litura* (Fabricius) (Lepidoptera: Noctuidae), is one of the most devastating pests of 120 crop plants, including cauliflower, groundnuts, cotton, onions, tomatoes, brinjal, turnips, and cabbage^2^. Each year, it goes through five to six overlapping generations, and if it is not promptly treated, it might cause significant crop losses up to complete destruction^3^. Insecticides are the most often used method for controlling this problem. Although this is effective in reducing pest populations in the short term, long-term exposure to insecticides may cause S*. litura* to develop the 3 R's issues, viz., resistance, resurgence of insects, and residues on crops, like other Noctuidae members^4^. In addition, the use of pesticides leads to ecological imbalances by destroying non-target organisms and their natural enemies, parasites, and predators. The public's growing concern over the potential ecological and health risks of synthetic pesticides has shifted the focus of research toward more environmentally benign methods for controlling insect pests^5^.

Insect-pathogenic or entomopathogenic fungi (EFP) (Fungi: Ascomycota, Order: Hypocreales) cause disease in insects. These entomopathogens are used as biocontrol agents, or "biopesticides," for the management of insect pests^6^. They provide an alternative to chemical insecticides for protecting crops^7^ and reducing the harmful environmental impacts of chemical insecticides^8^. An increasing number of products based on EPF are being registered as insecticides and used in developed and developing countries like the United States of America, the United Kingdom, Australia, Canada, China, India, etc.^8^.

Among the members of the genus Hypocreales, *Lecanicillium* sp., *Beauveria* sp., and *Metarhizium* sp. have been effectively used to control aphids, lepidopteron larvae, and other pests^9^. Among them, *Beauveria bassiana* (Balsamo) Vuillemin is responsible for causing white muscardine disease in a variety of insects. *Beauveria* infects the insect by degrading the host cuticle using mechanical and chemical forces, which are particularly advantageous in pest control because direct ingestion of fungal propagules is not needed by insects, thus also becoming active against the non-feeding stages of insects^10^. In addition, among the cyclic hexadepsipeptide mycotoxins produced by the different EPF, beauvericin, produced by *B. bassiana,* has shown the most effective larvicidal properties^11^.

Like other Hypocreales, the species of *Beauveria* show pleomorphic life stages. They are often described as cryptic fungi, i.e., morphological characteristics are changed in response to the environment, and thus morphological description fails to clarify their systematics at species level^12^. Nowadays, researchers are using polymerase chain reaction (PCR) based molecular techniques to reconstruct the *Beauveria* phylogeny for accurate identification of *Beauveria* species. Among the molecular markers, the internal transcribed spacer (ITS) region of rDNA is considered a universal bar code for fungus identification^13^. But in case of Hypocreales, ITS analysis produced low resolution in many cases and failed to resolve the phylogeny of *Beauveria*^14^*.* Additional genomic markers like translation elongation factor-1α (TEF) are needed for the species-level determination of *Beauveria* to be made correctly^14^.

Although *B. bassiana* showed a broad spectrum of pathogenicity against a wide range of insects, its bio-efficacy depends on the isolation source and life stages of the target stages. Insecticide resistance and resurgence issues can be effectively addressed by controlling insect pests with local isolates of fungus^15^. These native isolates also have higher survival and persistence abilities under local environmental conditions^16^. In conservation agriculture guidelines, it is also important to isolate potential native bioagents to prevent contamination from imported biopesticides. In addition, the pathogenicity of the biocontrol agent differs according to the different life stages of the target insect^17^. Identification of the more suspectable stage of insects against fungal inoculum increases the bio-efficacy of biological control strategies in field conditions. Therefore, the present investigation was carried out to isolate and molecularly characterize native *Beauveria* isolates and test their bio-efficacy against different life stages of *S. litura.*

## Results

### Isolates of *Beauveria*

**Figure 1:**
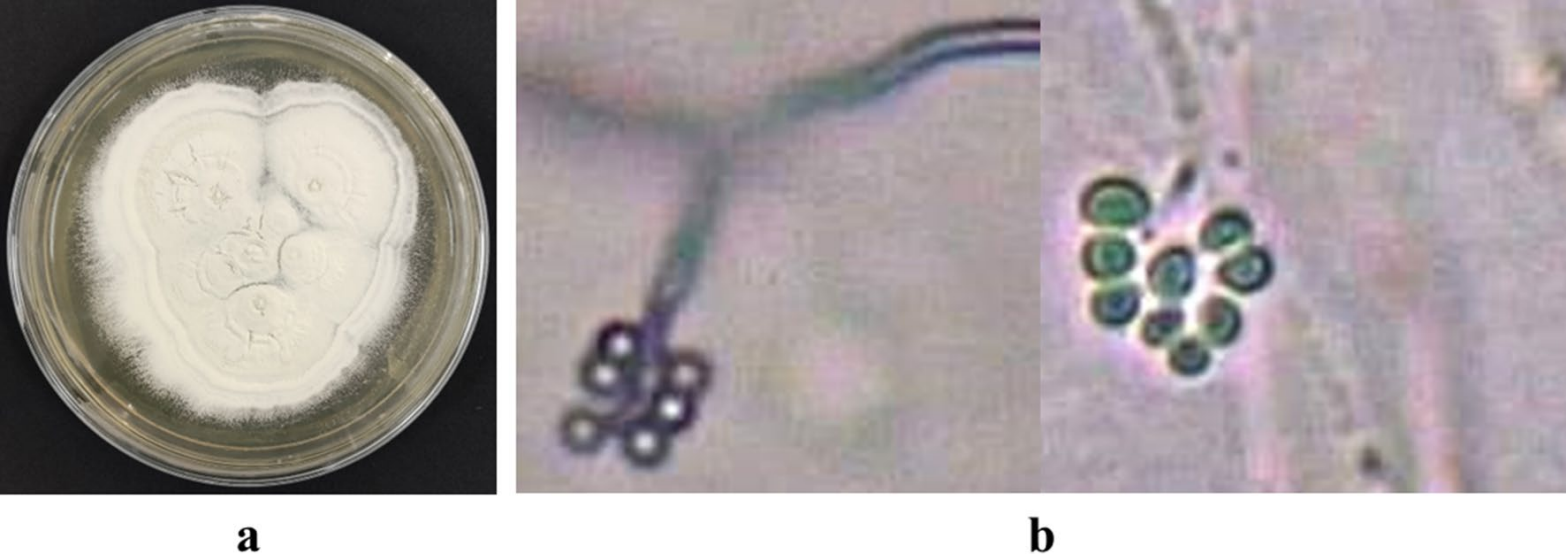
Morphological features of a representative *Beauveria* isolate TGS2.3. (**a**) 15 days old culture, (**b**) Conidiophore with conidia.

Among the isolated fungal isolates on selective medium, seven isolates showed characteristics of the morphology of *Beuaveria* species. The single fungal colonies of the isolates were white in color, round, lightly elevated with a powdery appearance, and lightly downy with circular rings. Conidia were hyaline and round. Single cell conidiophores were short, densely clustered, and hyaline (Fig. 1).

### Molecular identification and phylogeny of *Beauveria* isolates

**Figure 2:**
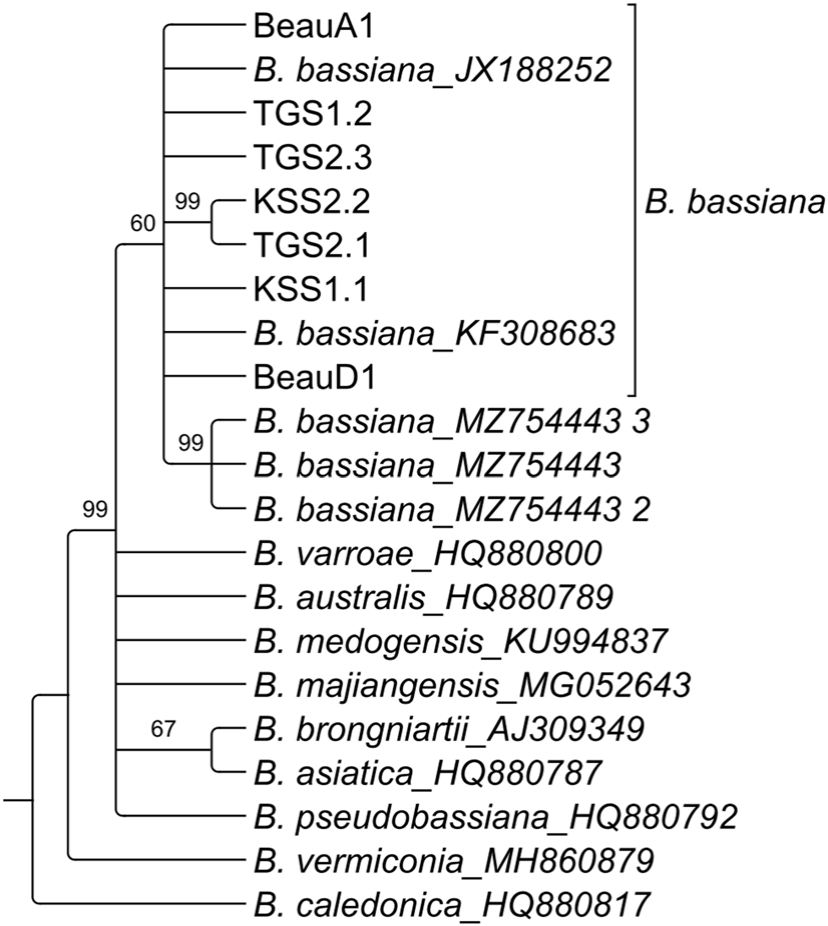
Maximum likelihood phylogenetic tree of the ITS data set of 1000 bootstrap replications in GTR-GAMMA model.

**Figure 3:**
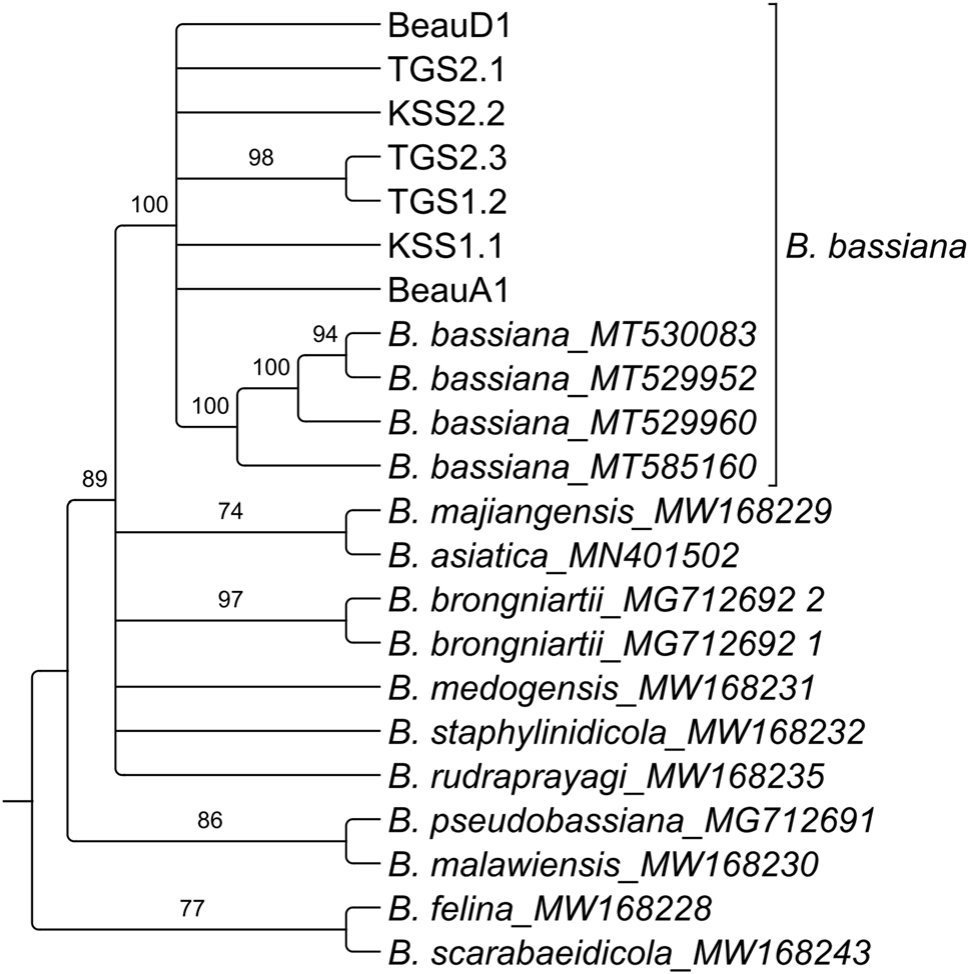
Maximum likelihood phylogenetic tree of the TEF data set of 1000 bootstrap replicates in GTR-GAMMA model.

The partial sequence datasets of ITS and TEF were processed and analyzed individually through Geneious V.11 software, and accession numbers were obtained from NCBI (Table 1). The genomic ITS and TEF data of seven isolated *Beauveria* isolates showed BLAST similarity, with many references to *B. bassiana* in BLAST search results in the NCBI database. The reconstructed maximum likelihood phylogenetic tree of the ITS data set showed that the seven morphologically characteristic isolates were clustered with the reference *B. bassiana* isolate with a moderate bootstrap support value (60%) (Fig. 2). Furthermore, a tree constructed with the TEF data set showed the maximum support (100%) for the clade containing isolated *Beauveria* isolates and references to *B. bassiana* (Fig. 3)*.* Thus, both the ITS and TEF data sets confirmed the isolated strains as *B. bassiana*.

**Table 1:** NCBI accession numbers of isolated *B. bassiana* isolates.

Serial no.	Isolate’s name	ITS accession	TEF accession
1	KSS1.1	OP784778	OP785280
2	KSS2.2	OP784779	OP785281
3	TGS1.2	OP784780	OP785282
4	TGS2.1	OP784781	OP785283
5	TGS2.3	OP784782	OP785284
6	BeauA1	OP784783	OP785285
7	BeauD1	OP784784	OP785286

### Biomass production of the fungal isolates

**Figure 4:**
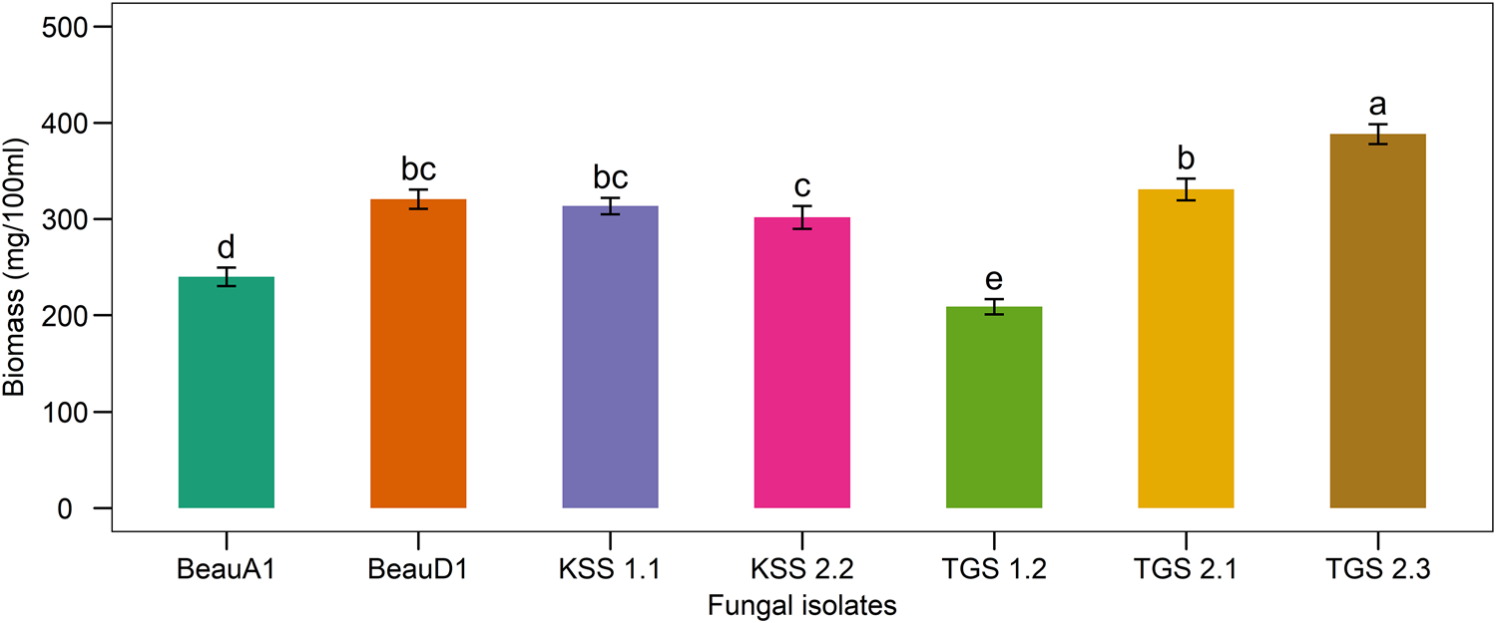
Biomass production of the *B. bassiana* isolates in SDA liquid broth. Values (means ± SEs) with different alphabetical letter(s) show statistically significant differences (lsd, p < 0.05).

Overall mean mycelial growth revealed that the fungal isolate TGS2.3 (388.27 ± 10.29 mg/100 mL) exhibited the highest biomass production, and the lowest growth was observed in TGS1.2 (208.8 ± 8.03 mg/100 mL) (Fig. 4).

### Insect bioassay

**Figure 5:**
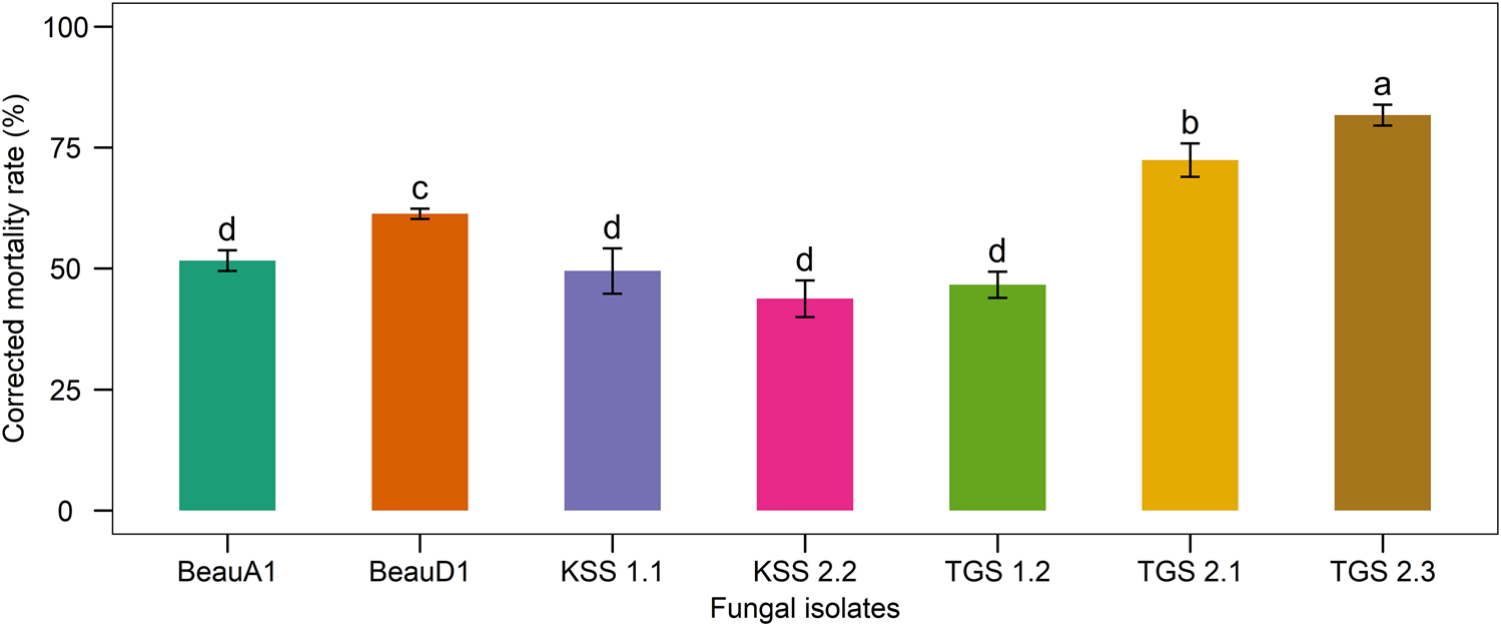
Mortality rates of 2nd instar larvae of *S. litura* treated with *Beauveria* isolates. Values (means ± SEs) with different alphabetical letter show statistically significant differences (lsd, p < 0.05).

Seven days following infection of the 2nd larval instar by seven *B. bassiana* isolates revealed that TGS2.3 had the highest mortality rates (81.72 ± 2.15%) followed by TGS2.1 (72.40 ± 3.46%), BeauD1 (61.29 ± 1.08%), BeauA1 (51.61 ± 2.15%), KSS1.1 (49.46 ± 4.69%), TGS1.2 (46.59 ± 2.71%), and KSS2.2 (43.73 ± 3.78%) (Fig. 5).

**Figure 6:**
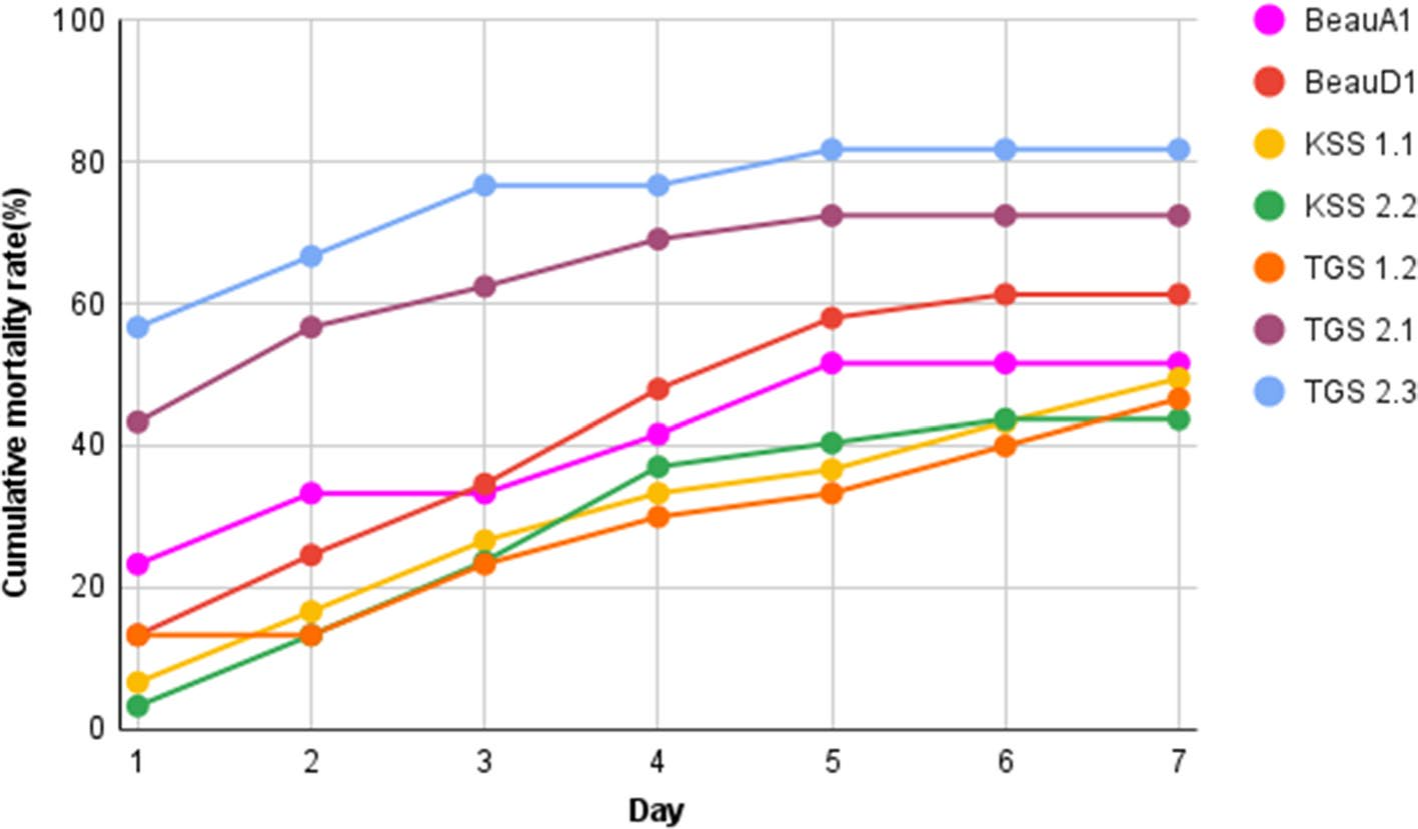
Cumulative mortality rates of *S. litura* treated with *B. bassiana* isolates over 7 days.

The findings implied that the death of 2nd instar larvae of *S. litura* treated with TGS2.3 and TGS2.1 occurred mostly during the first two days of infection, especially on the first day for TGS2.3. The mortality was caused more gradually from day-one to day-seven by the other *Beauveria* isolates, viz. BeauA1, BeauD1, KSS1.1, KSS1.2, KSS2.2, and TGS1.2 (Fig. 6).

**Figure 7:**
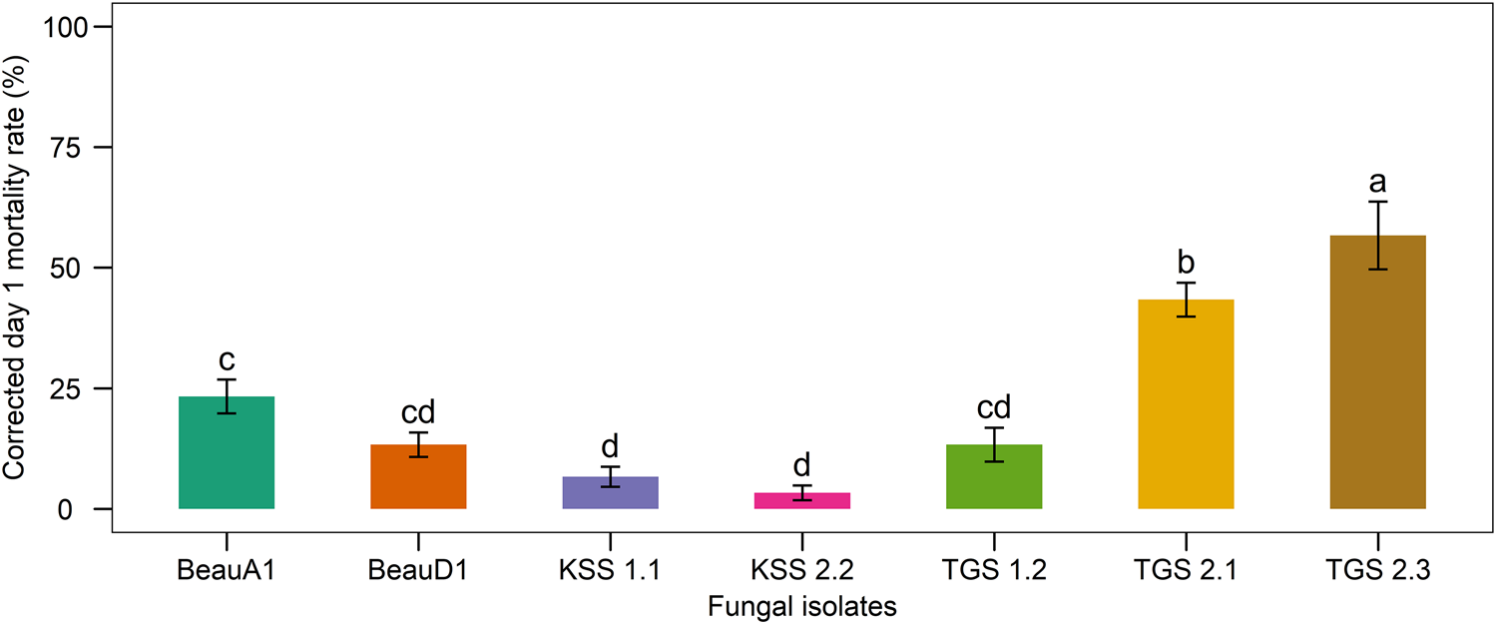
Day-one mortality of 2nd instar larvae of *S. litura* treated with *B. bassiana* isolates. Values (means ± SEs) with different alphabetical letter(s) show statistically significant differences (lsd, p < 0.05).

As the first day was when the most deaths occurred, results were statistically analyzed to ascertain which isolates induced the highest day-one mortality (causing high mortality within 24 h of infection). Samples infected with TGS2.3 (56.67 ± 7.02%) had the highest day-one mortality, followed by TGS2.1 (43.33 ± 3.51%) (Fig. 7).

### Hatchability and neonate larval mortality after TGS2.3 treatment

**Figure 8:**
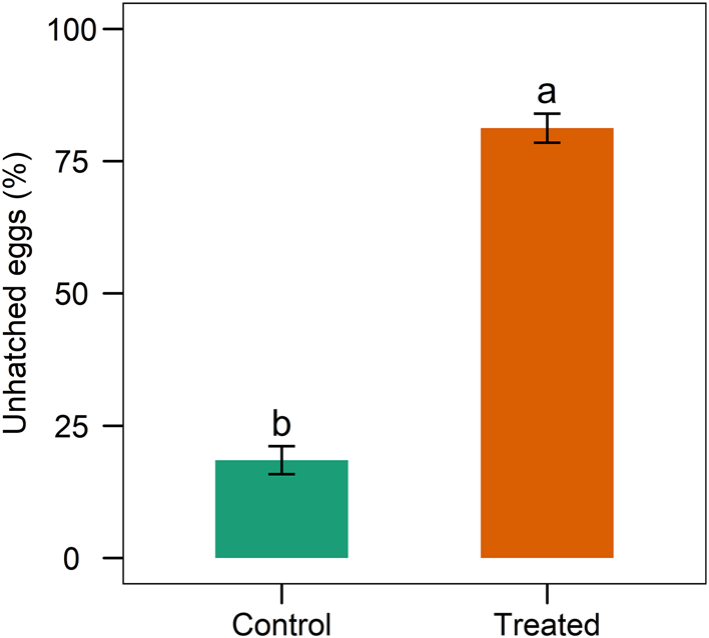
Mortality of *S. litura* eggs infected with *B. bassiana* isolate TGS2.3. Values (means ± SEs) with different alphabetical letter show statistically significant differences (lsd, p < 0.05).

**Figure 9:**
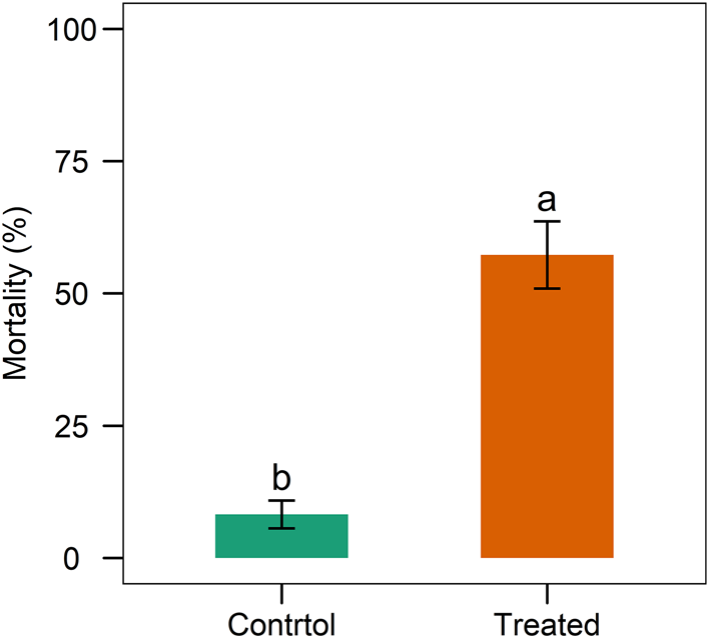
Mortality of neonate stage larvae treated with TGS2.3 at egg stage. Values (means ± SEs) with different alphabetical letter show statistically significant differences (lsd, p < 0.05).

Egg hatchability was drastically reduced in the TGS2.3-treated eggs compared to the control. The isolate TGS2.3 induced 81.25 ± 2.75% egg mortality, whereas in control it was 18.5 ± 2.65% (Fig. 8). The 7-days post treatment data also revealed that TGS2.3 induced 57.25 ± 6.34% neonatal larval mortality, whereas in control it was 8.25 ± 2.63% (Fig. 9).

### Bioassay against different larval stages of *S. litura* by *B. bassiana* isolate TGS2.3

**Figure 10:**
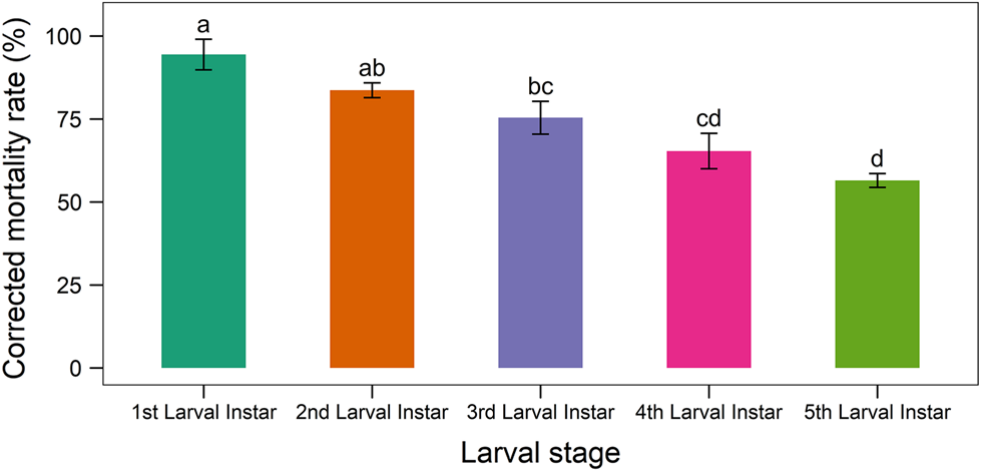
Mortality rates of different larval stages of *S. litura* treated with *B. bassiana* isolate TGS2.3. Values (means ± SEs) with different alphabetical letter(s) show statistically significant differences (lsd, p < 0.05).

The larvae treated with the TGS2.3 isolate succumbed to fungal infection and showed different mortality rates in various larval stages. The highest mortality was recorded in 1st instar larvae (94.45 ± 4.60%) and the lowest was in 5th instar larvae (56.56 ± 2.07%). The mortality rates in 3rd and 4th instar larvae were statistically similar (Fig. 10).

**Figure 11:**
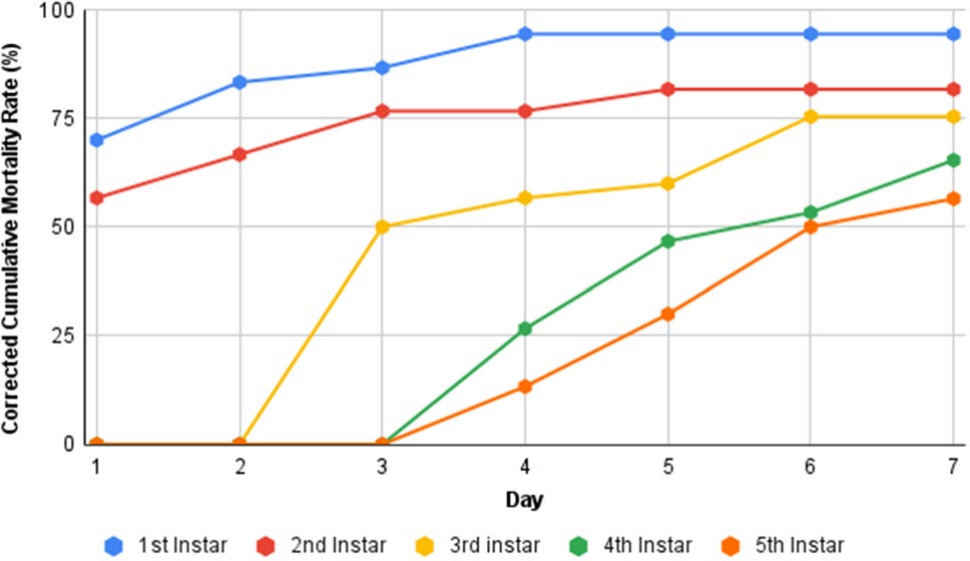
Cumulative mortality of different larval instar of *S. litura* treated with *B. bassiana* isolate TGS2.3.

Cumulative mortality over 7 days demonstrates that 1st instar larvae had the highest day-one mortality, which progressively rose until the 4th day. The death of 2nd instar larvae began on day-one and subsequently increased until day-five. The 3rd instar larvae did not die until the 3rd day, and the death rate progressed until the 6th day. The death of larvae in the 4th and 5th instars occurred on the 4th day and subsequently increased until the 7th day (Fig. 11). Overall, the mortality across various time points revealed all larval instars of *S. litura* to be susceptible to the fungus TGS2.3.

### Mycosis and sub-lethal effects

**Figure 12:**
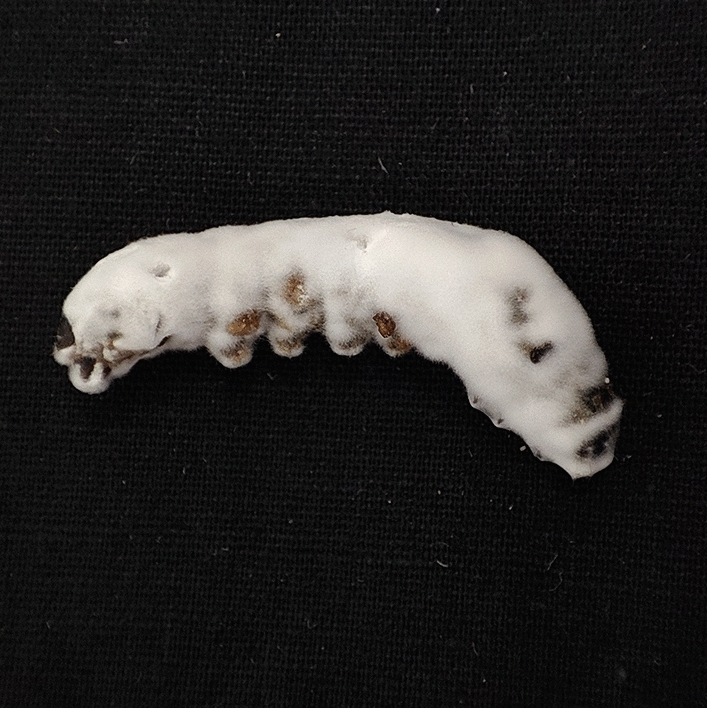
Larval cadaver of *S. litura* mycosed by *B. bassiana* isolate TGS2.3.

**Figure 13:**
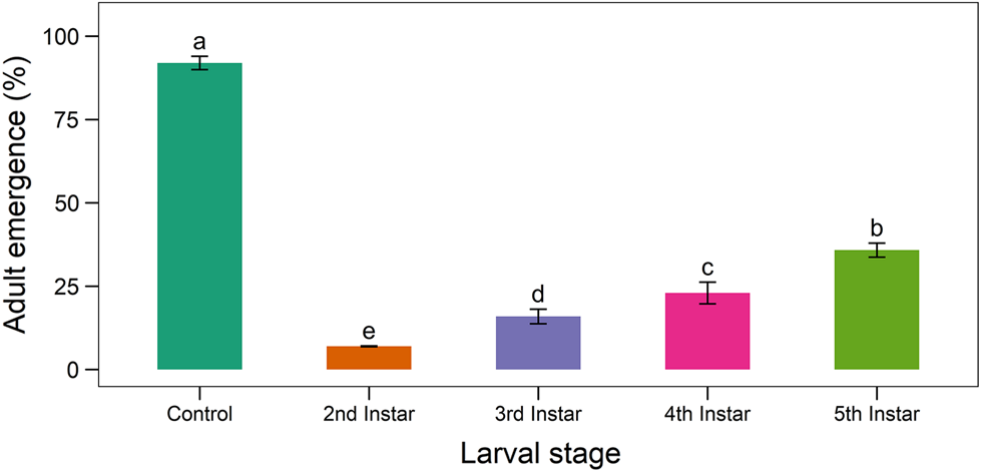
Adult emergence of larvae of *S. litura* due to treatment with *B. bassiana* isolate TGS2.3. Values (means ± SEs) with different alphabetical letter show statistically significant differences (lsd, p < 0.05).

The mobility of the infected larvae was reduced. The larvae were stiff and rigid after dying. Within two days of death, the deceased larvae began to produce mycelium. (Fig. 12). The *B. bassiana* infection was verified by the slides prepared from this fungus’ growth. When compared to control larvae, *B. bassiana* negatively impacted the emergence of adults from the 2nd, 3rd, 4th, and 5th instars. A smaller number of adults (7.11–37.94%) emerged from fungus treated larvae than from control larvae (94%) (Fig. 13).

### Deformities

**Figure 14:**
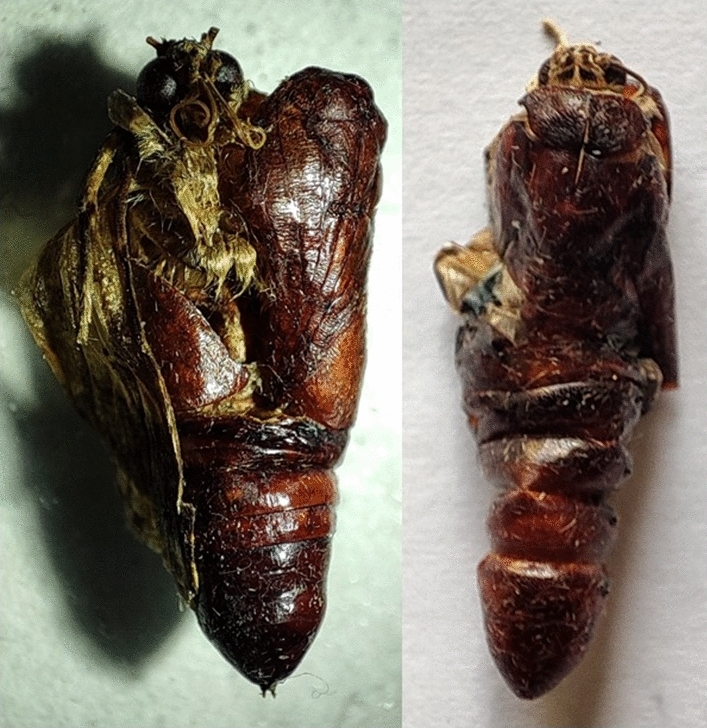
Undetached adult from exuvium.

**Figure 15:**
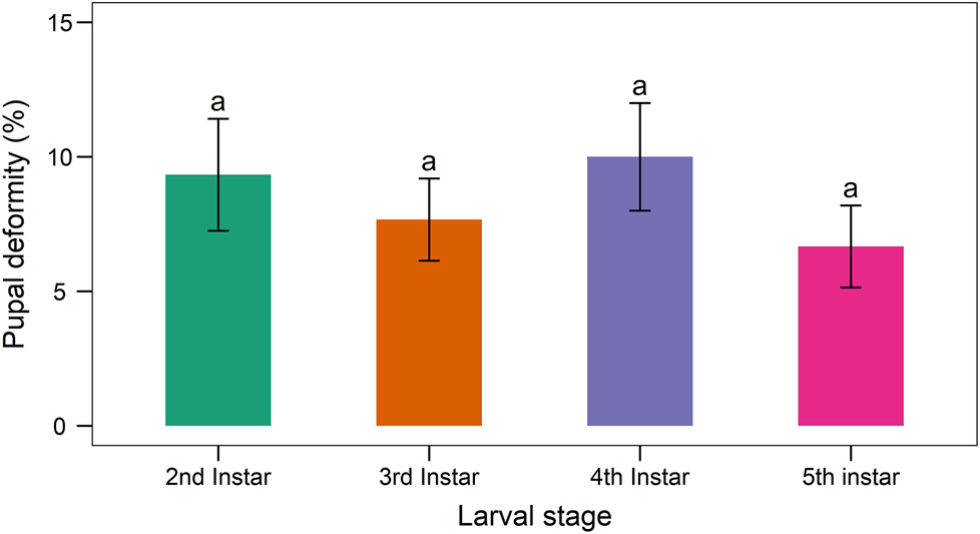
Pupal deformity of larvae of *S. litura* due to treatment with *B. bassiana* isolate TGS2.3. No statistically significant difference was observed among the values (means ± SEs) (lsd, p < 0.05).

**Figure 16:**
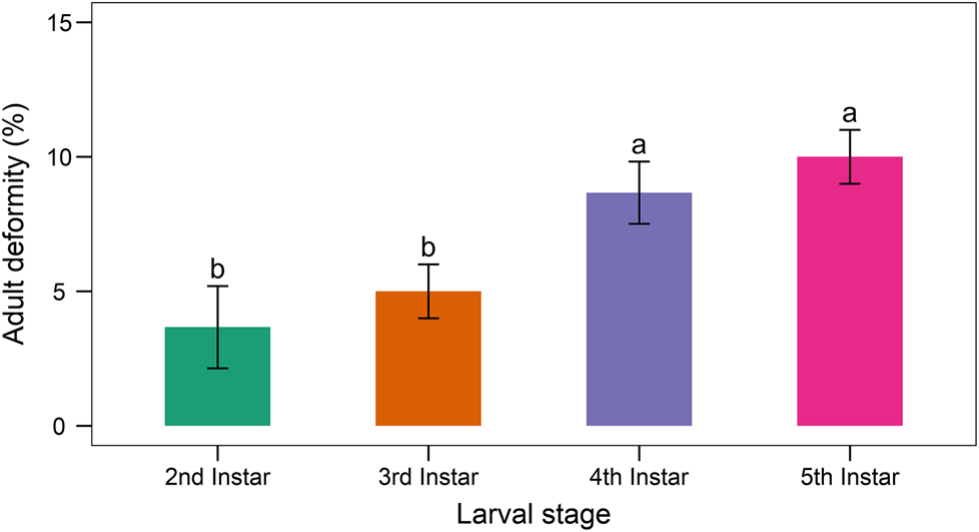
Adult deformity of larvae of *S. litura* due to treatment with *B. bassiana* isolate TGS2.3. Values (means ± SEs) with different alphabetical letter show statistically significant differences (lsd, p < 0.05).

**Figure 17:**
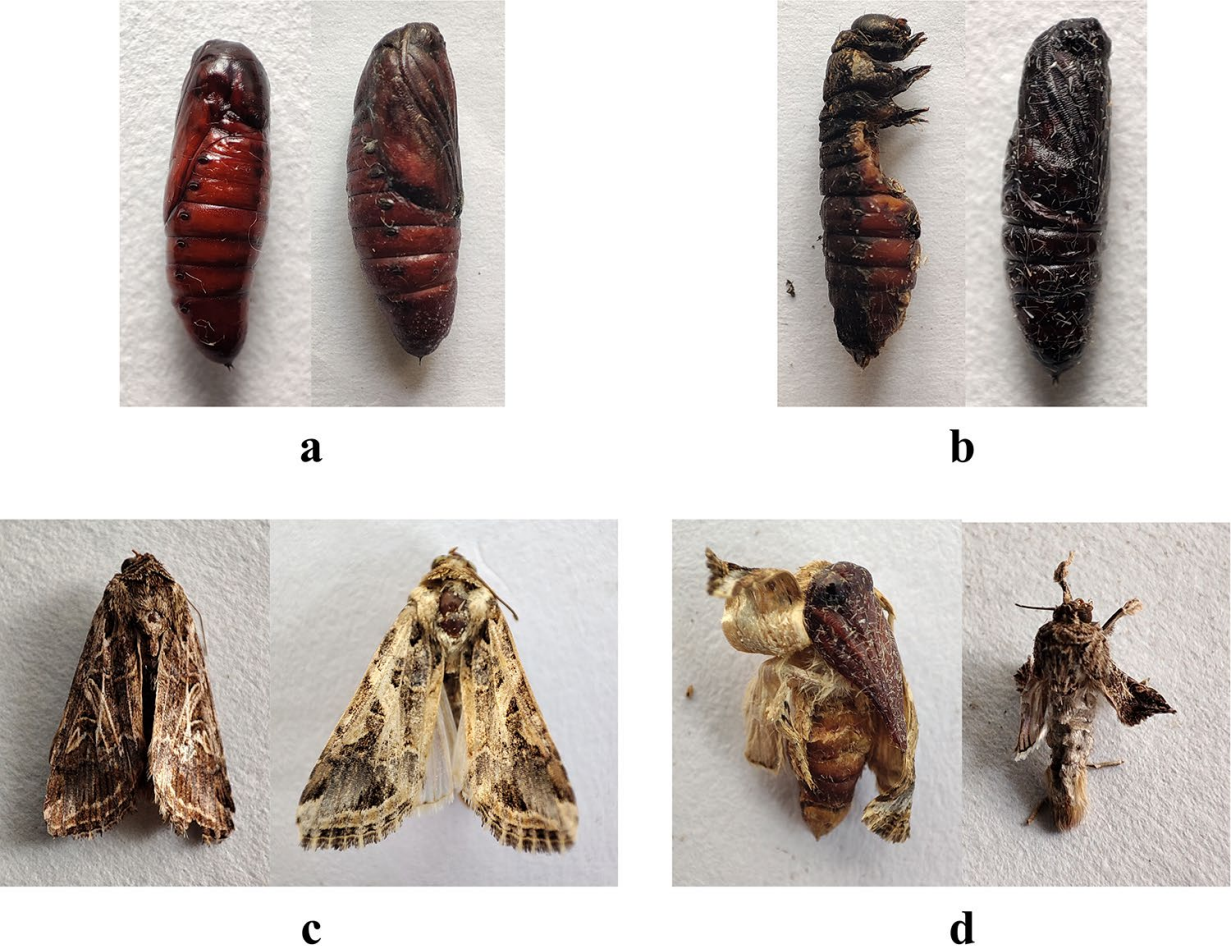
(**a**) Normal pupae, (**b**) Deformed pupae, (**c**) Normal adult, (**d**) Deformed adult.

The fungal infection caused a wide range of abnormalities. When some of the treated larvae molted into pupae, they did not entirely detach from the exuvium (Fig. 14). Some pupae lacked a completely developed cuticle. When 2nd instar larvae were treated with *B. bassiana*, they had 9.33 ± 2.08 percent pupal deformities. Similarly, the pupal deformity was 7.67 ± 1.53, 10 ± 2 and 6.67 ± 1.53 percent owing to the treatment of 3rd, 4th, and 5th instar larvae, respectively (Fig. 15). Adults developed from fungus infected larvae had 3.67–10% deformities (Fig. 16) with folded, undeveloped wings (Fig. 17). The control group, however, showed no deformation.

## Discussion

The tobacco caterpillar (*S. litura*) is one of the most devastating pests of various crops. Insecticides are the most commonly used method for controlling this problem. The use of pesticides leads to ecological imbalances by destroying non-target organisms and their natural enemies, parasites, and predators. The public's growing concern over the potential ecological and health risks of synthetic pesticides has shifted the focus of research toward more environmentally benign methods. Among them, *B. bassiana* causes white muscardine disease in a wide range of insects. Insecticide resistance and resurgence issues can be effectively addressed by controlling insect pests with local isolates of fungus and targeting more suspectable stages of insects.

In this study, seven *B. bassiana* isolates were isolated from soil samples and reported for the first time in Bangladesh as local isolates. The morphology described by previous studies^5,18^.was similar to that of our seven isolates. The ITS phylogeny produced a moderate support value for these seven isolates, which confirmed the inadequacy of the ITS analysis that had been previously reported^1,14,19^. However, ITS could be used for quick screening of a wide range of field isolates because of its PCR amplification efficiency^20–22^ and the availability of reference data^23^. Further molecular analysis with TEF data supported the phylogenetic position of seven isolates in the *B. bassiana* clade very strongly and proved its efficiency in resolving phylogenies for Hypocreales fungi^1,14,19^.

To find the best insect pathogenic *B. bassiana* isolate, the overall and daily mortality of 2nd instar larvae was investigated to determine the mortality induced by each fungal isolate. The highest mortality rate was induced by *B. bassiana* isolate TGS2.3 and could be because of higher insect pathogenic properties like conidial adhesion, germination rate, growth condition, or the production of enzymes or secondary metabolites. The very first stage of fungal infection is conidial attachment, and the strength of conidial attachment is a crucial indicator of the virulence of an entomopathogenic fungus. Fungal cell attachment to the cuticle may involve specific receptor-ligand and/or nonspecific hydrophobic and electrostatic mechanisms^24–26^. If the adhesion strength of EPF is weakened, then it could result in the washing off of the conidia from the host, thus preventing the infection^27^. The fluctuation of virulence among different isolates of *B. bassiana* may be due to their different levels of hydrophobic nature or their biochemistry.

Secondary metabolite synthesis might let EPF get past the immunological defenses of the insects and hasten mycosis^6^. According to some research, EPF *B. bassiana* creates host-specific secondary metabolites that, at low quantities, may result in 50% mortality^11,28^. The strain TGS2.3, which showed the highest insect mortality rate, may produce biologically active compounds with insecticidal activity against *S. liturta.* Further investigation is required to determine the bioactive compounds produced by *B. bassiana* isolate TGS2.3. The investigation and production of these compounds may open up new arrays of possibilities for controlling invasive crop pests.

The parameters, such as conidial germination and the production of hydrolytic enzymes are associated with the virulence of EPF^21,29–31^. A faster germination rate was found to exhibit higher virulence in *B. bassiana*^29^. The day-one mortality of TGS2.3 was the highest among all the isolates, which suggested that TGS2.3 has a higher germination rate. Hydrolytic enzymes such as protease, chitinase, and lipase are secreted by EPF to degrade the cuticle of host species to infect them^32^. Higher enzyme activity may be one of the reasons for the higher virulence of TGS2.3. Further investigation is needed to verify these hypotheses for our high-performing *Beauveria* candidate, TGS2.3.

The immobility of eggs is the main reason for insect vulnerability to microbial infections^33^. The nutrient requirement of an egg to develop into a hatchling is excessive, and they are highly targeted by pathogenic microbes at this stage^34^. This study showed that the eggs of *S. litura* were highly susceptible to TGS2.3. Similar results were found in previous studies where *B. bassiana* induced egg mortality in *S. frugiperda*^35,36^ and *Phthorimaea operculella*^37^. The isolate TGS2.3 also induced mortality in neonate larvae, which is similar to another study conducted by Idrees et al.^17^.

The mortality of larvae was highest in the 1st instar, and it gradually decreased with the advancement of each stage. The 1st instar larvae experienced 38% higher mortality than the 5th instar larvae. This indicates the decreased susceptibility of larvae with age. Shweta and Simon^38^ used *B. bassiana* against *S. litura* Fab. (Tobacco Caterpillar) in which the 1st and 2nd instar larvae showed higher mortality than the later stages. These variations in mortality between various instars might be attributed to enzymatic activity. According to different studies, detoxification enzyme activity changes significantly across and within developmental stages. The activity is modest in the egg stage, rises with each larval or nymphal instar, and ultimately decreases to zero during pupation^39,40^.

The EPF isolate TGS2.3 demonstrated sub-lethality over the life stages of *S. litura*. Pupal and adult deformities were produced as a consequence of the fungal infection in the larval stage. The larvae were unable to adequately transition into pupae. Insect molting has reportedly been hampered by *B. bassiana*^41^*.* Since the development of new cuticles during the molting process heavily depends on nutrients, any stage in the process might be affected if there is a nutritional imbalance in the hemolymph caused by a fungal infection. This sub-lethality of *B. bassiana* isolate TGS2.3 suggests a relatively prolonged sub-lethal influence of the fungi on *S.litura*, which may reduce *S.litura* populations more effectively in addition to direct mortality.

In summary, this study found that, the most potent isolate, TGS2.3, was effective against egg hatching and all stages of *S. litura* caterpillars and suggested that this fungal isolate could be utilized to target both the hatching and feeding stages of this target insect. Alongside, early stages of larval development of *S. litura* were more susceptible to fungal infection. The sub-lethal effects also demonstrated that once exposed to an entomopathogen, *B. bassiana* isolate TGS2.3 has the capability to kill insects at any descendant life stage of insect and reduce adult emergence. This study warrants further *in planta* evaluation in both laboratory and field conditions to evaluate the bio-efficacy of native *B. bassiana* isolates precisely. However, the findings of this research provided the potential for developing alternative *S. litura* pest control techniques as well as for limiting the use of synthetic pesticides, thereby minimizing their detrimental effects on the ecosystem.

## Methods

### Collection of soil samples

Soil samples were obtained from the Bhawal Sal Forest and agricultural fields in Gazipur, Bangladesh. To construct the composite sample, five different soil samples weighing a total of 250–300 g were mixed from a depth of 10–15 cm using a soil sampler. Until they were all studied, the soil samples were kept in distinct zip-lock bags with labels and maintained at 4 °C in a cold room.

### Isolation of fungus

A soil suspension containing five grams of soil and 50 mL of 0.1% Tween 80 was made in a screw-cap plastic tube and incubated at room temperature for 3 h after being sieved through a 5 mm screen. Five inversions of each tube were performed at intervals of 30 min. The tubes were retained for 20 s after incubation to allow for sedimentation, and 100 µL of supernatant from each tube was plated on a Petri plate with Sabouraud dextrose agar (SDA) medium (peptone 10 g/L, agar 10 g/L, dextrose 40 g/L, and CTAB 60 mg/L). To avoid bacterial contamination, streptomycin (30 mg/L) was also added. Following inoculation, all plates underwent a two-week incubation period at 22 °C. Every 2–3 days, plates were checked for recognizable, thick, compact white mycelium development. Hypocreales-like isolates were isolated and sub-cultured.

### Morphological study

Both the vegetative and reproductive structures of fungal colonies on SDA were examined using microscopy immediately after sporulation. From the outermost part of the fungal colony, little plaques were transferred to glass slides and inspected under a compound light microscope.

### Sub-culture, DNA isolation, and molecular characterization

On SDA agar plates without antibiotics, the fungal isolates were sub-cultured for DNA isolation and sequencing. The procedure described by Islam^1^ was used to extract the DNA.

Briefly, a little lump of fungal mycelium from a 7-day-old culture was placed in an Eppendorf tube, mashed with a sterile plastic pestle, and then suspended in 1 mL of lysis buffer (400 mM Tris–HCl, pH 8.0, 60 mM EDTA, 150 mM NaCl, and 1% SDS), which was then incubated at 50 °C for 1 h in a heat block. A volume of 150 μL of precipitation buffer (5 M potassium acetate, 60.0 mL; glacial acetic acid, 11.5 mL; distilled water, 28.5 mL) was added in the tube and vortexed shortly, then incubated on ice for 5 min. The supernatant from the centrifugation was transferred to a fresh tube along with 500 mL of isopropanol to precipitate the DNA. After centrifugation at 18,000 rcf for 20 min, the DNA pellet was recovered and washed with 1 mL of 70% ethanol. After being air dried for ten minutes, the DNA pellet was dissolved in 100 µL of Tris–EDTA (TE) buffer. In a nanodrop, the DNA's purity was examined. Polymerase chain reaction (PCR) was used to amplify the ITS region using the primers ITS1F: CTTGGTCATTTAGAGGAAGTAA and ITS4R: TCCTCCGCTTATTGATATGC in accordance with the profile: denaturation at 90 °C for 2 min, then 35 cycles of denaturation at 95 °C for 30 secs, annealing at 55 °C for 30 secs, extension at 72 °C for 1 min, and finally extension at 72 °C for 15 min^1^. The 5′-TEF region was amplified using EF1TF (5′-ATGGGTAAGGARGACAAGAC) and EF2TR (5′-GGAAGTACCAGTGATCATGTT) after the profile underwent an initial denaturation at 94 °C for 5 min, followed by 35 cycles at 94 °C for 40 s, 65 °C for 40 s, 72 °C for 2 min, and a final extension at 72 °C for 10 min^19^. The PCR product was electrophoresed in 1% agarose in 1 × TBE buffer at 120 V with GelRed nucleic acid stain and photographed with a molecular imager under UV light. For sequencing, the PCR products were sent to Macrogen, Korea.

### Sequence analysis and phylogenetic tree preparation

The Sanger sequencing data of the fungal isolates were produced, and a BLAST search on the National Center for Biotechnology Information (NCBI) database was completed. The partial sequence datasets of ITS and TEF were submitted to NCBI for getting accession number. The sequences matched reference genome sequences obtained from NCBI. The Geneious V.11's MAFT plug-in was used for multiple alignments, and the final alignment was fixed manually. Phylogenetic trees were developed by maximum likelihood analysis for the data sets using the Geneious V.11 RAxML plug-in using rapid bootstrapping and searching for the best scoring ML tree from 1000 bootstrap replicates in the GTR-GAMMA model.

### Insect rearing

Eggs of *S. litura* were obtained from the existing culture at the Entomology Division, Bangladesh Agricultural Research Institute (BARI), Gazipur, Bangladesh. Homogenous larvae were obtained from eggs hatched on the same day. The larvae were grown in sterile plastic boxes containing pieces of okra disinfected with 0.5% (v/v) sodium hypochlorite for 10 min, maintained at 25 ± 2 °C and 65 ± 5% RH^42^.

### Production and Collection of *Beauveria* conidia

Sabouraud’s Dextrose Agar (SDA) medium was used in this study. A 10 mm actively grown culture of *B. bassiana* was placed individually at the center of the 60 mm petri dish containing 10 mL of solid SDA media^43^. The inoculated plates were incubated at 28 ºC for 7 days. The conidial suspension of the isolates was then prepared by flooding the dishes with 10 mL of sterile Tween 80 (0.05%), the agar surface was gently scraped with sterile glass rods, and the suspension was collected in sterile 250 mL beakers. The suspension was then adjusted to 50 mL and mixed using a hand mixer to separate and disperse the conidia, and finally the conidial density was adjusted to 1.5 × 10^8^ conidia per ml using a hemocytometer^44^. Before the bioassay experiment, conidial germination was tested on SDA agar medium.

### Growth in liquid medium

A volume of 250 mL Sabouraud’s Dextrose Broth (SDB) was prepared in a 500 mL Schott bottle, and the final pH was adjusted to 6.5. The liquid broths were then inoculated with a 10 mm culture disc of the fungus. Three replications were maintained for all the *B. bassiana* isolates. The entire setup was kept in a shaker incubator at 25 °C temperature at 120 rpm for 10 days. White cotton ball-type growth was observed after 7 days. The mycelia were then filtered through a pre-weighed filter paper and dried in a hot air oven at 70 °C until a constant weight was obtained. This revealed the biomass production capability of all the fungal isolates ^43^.

### Virulence of *B. bassiana* isolates against eggs and hatched larvae

Freshly laid egg masses that were 1–2 days old were collected and counted under a dissecting microscope. A batch of 50 eggs was separated using a hairbrush and transferred into a petri dish. A volume of 10 mL of conidial suspension (1.5 × 10^8^ conidia/mL) was made using 0.05% Tween 80. The suspension was then sprayed over the egg masses. For control, only Tween 80 was used. Each treatment was repeated four times. 7 days after the treatment (DAT), the number of hatched and unhatched eggs was counted. The newly hatched larvae were then fed, incubated at 25 ± 2 °C, and monitored for the next 7 days. The mortality of each treatment was carefully recorded^17^.

### Insect bioassay

Freshly laid eggs were collected and hatched to obtain homogenous larvae. The assay was conducted on 2nd instar larvae of* S. litura.* A set of 10 larvae in triplicate were dipped individually into a 10-mL conidial suspension of *Beauveria* isolates (1.5 × 10^8^ conidia/mL) for 5 s. After treatment, transferred each set of larvae to a separate, sterile plastic box. To each box, added moist blotting paper and a piece of disinfected okra as feed. Changed the paper and feed on alternate days*.* At 7 DAT, the mortality of larvae was recorded according to the isolates^42^.

### Evaluation of sublethal effects

Larvae that survived the fungal treatment were further reared until pupation at 25 ± 2 °C and 60–70% relative humidity to see the sublethal activity, such as variation in development, any kind of deformity, and longevity compared to the control. Observations were made on larval and pupal deformity, adult emergence, and any morphological deformity in various developmental stages^3^.

### Statistical analysis

Mortality was corrected by Abbott’s formula^45^. The percent data were transformed by the arcsine transformation. The data were subjected to an analysis of variance (ANOVA), followed by a comparison of the means of different treatments using the least significant difference (LSD). Analyses were performed using R version 3.4.2.

## Data Availability

The partial sequence data of ITS and TEF genomic regions of fungal isolates during the current study are available in the NCBI repository under the Accession Numbers OP784778–OP784784 and OP785280–OP785286 (will be available on December 4 2022), respectively. The statistical datasets used and/or analyzed during the current study are available from the corresponding author upon reasonable request.

## References

[1] Islam SMN (2018). Systematics, Ecology and Plant Associations of Australian Species of the Genus Metarhizium.

[2] Dhanapal R, Kumar D, Lakshmipathy R, Rani CS, Kumar VM (2020). Isolation of indigenous strains of the white halo fungus as a biological control agent against 3rd instar larvae of tobacco caterpillar, *Spodoptera litura* (Fabricius) (Lepidoptera: Noctuidae). Egypt. J. Biol. Pest Control.

[3] Kaur S, Kaur HP, Kaur K, Kaur A (2011). Effect of different concentrations of *Beauveria bassiana* on development and reproductive potential of *Spodoptera litura* (Fabricius). J. Biopest..

[4] Mkenda PA (2020). Knowledge gaps among smallholder farmers hinder adoption of conservation biological control. Biocontrol Sci. Tech..

[5] Dhar S, Jindal V, Jariyal M, Gupta V (2019). Molecular characterization of new isolates of the entomopathogenic fungus *Beauveria bassiana* and their efficacy against the tobacco caterpillar, *Spodoptera litura* (Fabricius) (Lepidoptera: Noctuidae). Egypt. J. Biol. Pest Control.

[6] Zimmermann G (2007). Review on safety of the entomopathogenic fungus *Metarhizium anisopliae*. Biocontrol Sci. Tech..

[7] Lacey L, Goettel M (1995). Current developments in microbial control of insect pests and prospects for the early 21st century. Entomophaga.

[8] Kabaluk JT, Svircev AM, Goettel MS, Woo SG (2010). The Use and Regulation of Microbial Pesticides in Representative Jurisdictions Worldwide.

[9] Quesada-Moraga E, Navas-Cortés JA, Maranhao EA, Ortiz-Urquiza A, Santiago-Álvarez C (2007). Factors affecting the occurrence and distribution of entomopathogenic fungi in natural and cultivated soils. Mycol. Res..

[10] Bateman R, Douro-Kpindou O, Kooyman C, Lomer C, Ouambama Z (1998). Some observations on the dose transfer of mycoinsecticide sprays to desert locusts. Crop Prot..

[11] Wang Q, Xu L (2012). Beauvericin, a bioactive compound produced by fungi: A short review. Molecules.

[12] Imoulan A, Hussain M, Kirk PM, El Meziane A, Yao Y-J (2017). Entomopathogenic fungus *Beauveria*: Host specificity, ecology and significance of morpho-molecular characterization in accurate taxonomic classification. J. Asia-Pac. Entomol..

[13] Kõljalg, U. *et al.* (Wiley, 2013).

[14] Rehner SA (2011). Phylogeny and systematics of the anamorphic, entomopathogenic genus *Beauveria*. Mycologia.

[15] Goble T, Dames J, Hill M, Moore S (2011). Investigation of native isolates of entomopathogenic fungi for the biological control of three citrus pests. Biocontrol Sci. Tech..

[16] Sain SK (2019). Compatibility of entomopathogenic fungi with insecticides and their efficacy for IPM of *Bemisia tabaci* in cotton. J. Pestic. Sci..

[17] Idrees A, Afzal A, Qadir ZA, Li J (2022). Bioassays of Beauveria bassiana isolates against the fall armyworm, *Spodoptera frugiperda*. J. Fungi.

[18] Glare TR, Inwood AJ (1998). Morphological and genetic characterisation of *Beauveria* spp. from New Zealand. Mycol. Res..

[19] Bischoff JF, Rehner SA, Humber RA (2009). A multilocus phylogeny of the *Metarhizium anisopliae* lineage. Mycologia.

[20] White TJ, Bruns T, Lee S, Taylor J (1990). Amplification and direct sequencing of fungal ribosomal RNA genes for phylogenetics. PCR Protoc..

[21] Heale JB, Isaac JE, Chandler D (1989). Prospects for strain improvement in entomopathogenic fungi. Pestic. Sci..

[22] Vilgalys R, Gonzalez D (1990). Organization of ribosomal DNA in the basidiomycete *Thanatephorus praticola*. Curr. Genet..

[23] Lutzoni F (2004). Assembling the fungal tree of life: Progress, classification, and evolution of subcellular traits. Am. J. Bot..

[24] Boucias D, Pendland J, Latge J (1988). Nonspecific factors involved in attachment of entomopathogenic deuteromycetes to host insect cuticle. Appl. Environ. Microbiol..

[25] Boucias DG, Pendland JC (1991). The Fungal Spore and Disease Initiation in Plants and Animals.

[26] Doss RP, Potter SW, Chastagner GA, Christian JK (1993). Adhesion of nongerminated *Botrytis cinerea* conidia to several substrata. Appl. Environ. Microbiol..

[27] Holder DJ, Keyhani NO (2005). Adhesion of the entomopathogenic fungus *Beauveria* (Cordyceps) *bassiana* to substrata. Appl. Environ. Microbiol..

[28] Quesada-Moraga E, Alain V (2004). Bassiacridin, a protein toxic for locusts secreted by the entomopathogenic fungus *Beauveria bassiana*. Mycol. Res..

[29] Faria M, Lopes RB, Souza DA, Wraight SP (2015). Conidial vigor vs. viability as predictors of virulence of entomopathogenic fungi. J. Invertebr. Pathol..

[30] Petrisor C, Stoian G (2017). The role of hydrolytic enzymes produced by entomopathogenic fungi in pathogenesis of insects mini review. Roman. J. Plant Prot..

[31] Tseng M-N, Chung C-L, Tzean S-S (2014). Mechanisms relevant to the enhanced virulence of a dihydroxynaphthalene-melanin metabolically engineered entomopathogen. PLoS ONE.

[32] Cheong P, Glare TR, Rostás M, Haines SR (2016). Microbial-Based Biopesticides.

[33] Tillman P (2010). Parasitism and predation of stink bug (Heteroptera: Pentatomidae) eggs in Georgia corn fields. Environ. Entomol..

[34] Kellner, R. L. The role of microorganisms for eggs and progeny. in *Chemoecology of insect eggs and egg deposition*, 149–164 (2002).

[35] Cruz-Avalos AM, Bivián-Hernández MDLÁ, Ibarra JE, Del Rincón-Castro MC (2019). High virulence of Mexican entomopathogenic fungi against fall armyworm, (Lepidoptera: Noctuidae). J. Econ. Entomol..

[36] Idrees A (2021). Effectiveness of entomopathogenic fungi on immature stages and feeding performance of Fall Armyworm, *Spodoptera frugiperda* (Lepidoptera: Noctuidae) Larvae. Insects.

[37] Khorrami F, Mehrkhou F, Mahmoudian M, Ghosta Y (2018). Pathogenicity of three different entomopathogenic fungi, *Metarhizium anisopliae* IRAN 2252, *Nomuraea rileyi* IRAN 1020C and *Paecilomyces tenuipes* IRAN 1026C against the potato tuber moth, *Phthorimaea operculella* Zeller (Lepidoptera: Gelechiidae). Potato Res..

[38] Shweta A, Simon S (2017). Efficacy of *Beuveria bassiana* on different larval instars of tobacco caterpillar (*Spodoptera litura* Fab.). Int. J. Curr. Microbiol. Appl. Sci..

[39] Ahmad S (1986). Enzymatic adaptations of herbivorous insects and mites to phytochemicals. J. Chem. Ecol..

[40] Mullin CA (1988). Adaptive relationships of epoxide hydrolase in herbivorous arthropods. J. Chem. Ecol..

[41] Torrado-León E, Montoya-Lerma J, Valencia-Pizo E (2006). Sublethal effects of *Beauveria bassiana* (Balsamo) Vuillemin (Deuteromycotina: Hyphomycetes) on the whitefly *Bemisia tabaci* (Gennadius) (Hemiptera: Aleyrodidae) under laboratory conditions. Mycopathologia.

[42] Tupe SG, Pathan EK, Deshpande MV (2017). Development of *Metarhizium anisopliae* as a mycoinsecticide: From isolation to field performance. JoVE.

[43] Senthamizhlselvan P, Sujeetha JAR, Jeyalakshmi C (2010). Growth, sporulation and biomass production of native entomopathogenic fungal isolates on a suitable medium. J. Biopest..

[44] Zhang S, Gan Y, Xu B, Xue Y (2014). The parasitic and lethal effects of *Trichoderma longibrachiatum* against *Heterodera avenae*. Biol. Control.

[45] Abbott WS (1925). A method of computing the effectiveness of an insecticide. J. Econ. Entomol.

